# Fetal macrosomia and its associated factors among singleton live-births in private clinics in Mekelle city, Tigray, Ethiopia

**DOI:** 10.1186/s12884-019-2379-3

**Published:** 2019-07-01

**Authors:** Freweini Gebrearegay Tela, Afework Mulugeta Bezabih, Amaha Kahsay Adhanu, Kidanemariam Berhe Tekola

**Affiliations:** Department of Nutrition and Dietetics, School of Public Health, College of Health Sciences, Mekelle University, Tigray, Ethiopia

## Abstract

**Background:**

Despite an increased number of infants born with macrosomia globally, low birth weight infants have currently attracted more attention. Macrosomia is a growing problem in most developing countries and it directly or indirectly contributes to morbidity, mortality, and disability worldwide. The main objective of this study was to assess the level of macrosomia and its associated factors in the private clinics of Mekelle city, Tigray region, Ethiopia, 2017.

**Methods:**

An institution based cross-sectional study with a total of 309 pregnant mothers was conducted. We collected data from the pregnant mothers as well as from their medical records using structured questionnaire and checklist respectively. We entered and analyzed the data using statistical package for social science (SPSS)-21 by applying binary logistic regression to identify the factors associated with macrosomia. Finally, we used texts and tables to summarize the results of the study.

**Results:**

The prevalence of macrosomia was 19.1% (95% confidence interval (CI) = 14.9, 23), and the mean ± standard deviations of birth weights were 3440 ± 543 g. Macrosomia was significantly associated with: weight gain during pregnancy ≥16 kg (adjusted odds ratio (AOR) = 11, 95% CI: 3, 37), pre-pregnancy overweight (AOR = 5, 95% CI = 2, 13), pre-pregnancy obesity (AOR = 15, 95% CI = 5, 50), maternal age (AOR =2.6, 95% CI = 1.2, 5.8) and giving birth to macrosomic baby in the last pregnancy (AOR = 2.7, 95% CI = 1.1, 7).

**Conclusion:**

We found that prevalence of macrosomia was high, and significantly associated with pre-pregnancy body mass index (BMI), pregnancy weight gain, maternal age and giving birth to a macrosomic baby in the last pregnancy. Hence, we recommend that emphasis should be given to maternal counseling for weight management before and during pregnancy.

## Background

It is increasingly recognized that the influence of racial variation in birth weight (BW) is substantial, and there is no common consensus among researchers on the definition of macrosomia. Most of the researchers from low and middle-income countries define macrosomia as a BW of 4000 g or more for singleton pregnancies [1–4]. Despite an increased number of infants born with macrosomia globally, low birth weight infants have currently attracted more attention [2]. Currently, macrosomia is a growing problem in most developing countries and it directly or indirectly contributes to morbidity, mortality, and disability worldwide [3, 4]. It is also evidenced that being born macrosomic is associated with health risks in later life, as the concept of fetal onset of adult disease hypothesis described that both growth-restricted and macrosomic infants are highly predisposed to coronary artery disease, hypertension, obesity, and insulin resistance in adulthood [1, 5].

Besides the risk of mortality and morbidity for the mother, macrosomia may have many other maternal complications including dysfunctional uterine contraction, prolonged labor, cesarean delivery, uterine rupture, nerve injury, and lower genital tract lacerations. It may also have neonatal complications such as clavicular or humerus fracture, shoulder dystocia, brachial plexus injuries, neonatal asphyxia, hypoglycemia, increased risk of neonatal infection and perinatal death [2, 6–10].

Factors associated with macrosomia may include but are not limited to, maternal age, pre-pregnancy BMI, weight gain during pregnancy, prior history of diabetes, previous macrocosmic delivery, gestational age, and parity [1, 6, 8–13]. Its prevalence is also believed to be higher in industrialized nations and among women of high socioeconomic status within a given population [1]. Identifying the risk factors associated with macrosomia at the local level is of high importance to take appropriate measures during the antenatal period to reduce its prevalence, eventually reducing the prevalence of the aforementioned complications. Therefore, the purpose of this study was to determine the prevalence of macrosomia and investigate the associated risk factors in Mekelle city, Tigray, Ethiopia.

## Methods

### Study design

We applied a health facility-based cross-sectional study design to assess the prevalence and associated factors of macrosomia.

### Study area and period

The study area, Mekelle city, is located in Tigray regional state found in the northern part of Ethiopia. Tigray region is bordered by Eritrea in the North, Sudan to the West, Amhara regional state of Ethiopia in the Southwest and Afar region of Ethiopia to the East. Mekelle city is 780 km far from the capital city of the country, Addis Ababa, and has a total population of 358,529 with an estimated number of pregnant mothers of 12,333 by the year 2016/17. Data were collected between April 1st and May 22nd, 2017.

### Sources and study population

All of the pregnant mothers who have attended antenatal care (ANC) services from private clinics in Mekelle city were the target population, and pregnant mothers who attend ANC from the selected clinics during the study period were the study population.

### Inclusion and exclusion criteria

We included mothers with a singleton pregnancy. But, mothers who were excluded from this study were those with current or chronic medical illnesses such as diabetes and hypertension, and those with multiple (two or more) pregnancies (as determined using ultrasonography).

### Sample size calculation

We used a single population proportion formula using Open-epi version 2.3 software package to determine the sample size for this study [17], we considered 95% CI and 0.05 margin of error to calculate this sample size. We took the proportion of macrosomia (P), 15.8%, from a study [14], and got a sample size of 206. Multiplying this sample size by 1.5 (design effect), the final sample size became 309.

### Sampling techniques

From the clinics with a relatively higher caseload (serving ≥30 pregnant mothers per month), we selected five private clinics at random. Following this, we selected mothers proportionally from each selected clinic using the flow of pregnant mothers to the clinics in the last three months before the start of the data collection as a baseline. Finally, the mothers coming to the clinics were consecutively taken until the sample size was attained.

### Study variables

We designed the tool to have four parts. These include: socio-demographic characteristics (maternal age, religion, marital status, educational status, occupational status, family size, residence), obstetric factors (parity, length of gestation, inter-pregnancy interval, the sex of the newborn, and birth weight of the last child), anthropometric measurements (maternal weight, maternal height, weight of the newborn etc.), and behavioral factors (physical activity, smoking status, alcohol drinking, and feeding practice (dietary diversity score)). We also assessed birth weight as a dependent variable.

### Data collection methods and tools

Data were collected during the early pregnancy (around 12 weeks) using an interviewer-administered structured questionnaire and checklist. The tools were administered by trained midwives of the clinics and cross-checked by the principal investigator to ensure its completeness. The weight of the pregnant mothers was measured during their first antenatal care (ANC) visit using a weight measuring scale and recorded accurate to 100 g, and this continued in every visit. The measurements were made on the participants wearing of a minimum amount of clothing, and calibration was made after every two measurements. Maternal weight gain during pregnancy was estimated by subtracting the early first-trimester weight from the last measured weight before delivery. The newborns were weighed within 2 h of birth using a beam balance accurate to 100 g. The height of the mothers was measured using a calibrated height measuring steel attached to the beam balance and recorded accurate to 0.1 cm (cm). The mothers were asked to maintain an upright and erect position with their feet together, and a horizontal headpiece was lowered onto the women’s head.

A single 24-h dietary recall method was utilized to calculate the women’s dietary diversity score (WDDS). A category of nine food groups was made from all of the foods and fluids that were consumed before the date of the data collection by the study participants. For those who consumed an item from any of the food groups, a score of one was given; and a score of zero was assigned if no food was taken. Eventually, by adding up all of the food groups consumed by the study participants in the last 24 h, we classified them as having low WDDs (if ≤3 food groups were consumed), medium WDDs (if 4–6 food groups were consumed) and high WDDs (if 7–9 food groups were consumed) [14].

### Data quality assurance

To ensure the quality of data, a carefully designed data collection tool was prepared. A common understanding of the data collectors and supervisors of the overall study was maintained by providing two days of training. The questionnaire was pre-tested before actual data collection, and modifications were made accordingly. The questionnaire was translated to the local language (Tigrigna), then back-translated to English to attain consistency. The collected data were checked by the supervisors periodically for its accuracy and completeness, and it was cross-checked and cleaned by the principal investigator before and after the data entry.

### Data management and analysis

After coding the questionnaire, data were entered and analyzed using SPSS-21 computer software package. Simple frequency tables were used for sorting, tabulating and cleaning of the data. Bivariate analysis with 95% confidence interval was used to infer association, controlling the effect of confounding factors using a multivariable logistic regression model. Significance was determined using the adjusted and unadjusted odds ratio with 95% CI and *p*-value. Variables with a *p*-value < 0.05 in multivariable analysis were declared as statistically and independently significant predictors of macrosomia. The Hosmer-Lemeshow goodness of fit test provided evidence for model fit with the predictors (*p* = 0.673). Multicollinearity effect was checked in the multivariable regression model, and all the variables had a standard error < 2.0.

### Ethical consideration

Ethical approval was obtained from the of the College of Health Sciences of Mekelle University institutional review board (IRB) before the study was conducted. Permission was sought from the private clinics, and the study participants provided verbal consent after being informed about the overall purpose of the study. We maintained the confidentiality of the participants throughout the study, and their right to participate, refuse or stop was guaranteed at any time of the data collection period. It was explained to the participants before the data collection that the procedures of this study have minimal risk.

## Results

### Socio-demographic characters of the study participants

A total of 309 participants was included in this study, with a 100% response rate. Almost all (99.7%) of the mothers were urban residents, and 3/4th were housewives. The mean age of the mothers was 28.5 ± 3.9 years, and 58.6% of them were < 30 years old. Regarding the educational status of the mothers, three-fifths (60%) of them were diploma and above (Table 1). Almost all (99%) of the mothers have never smoked, and the majority (89%) have never drunk alcohol during their current pregnancy.

**Table 1:** Socio-demographic characteristics of the study participants (309)

Variables	Categories	Frequency	Percentage
Age of the mother (years)	< 30 years	181	58.6
	≥30 years	128	41.4
The religion of the mother	Orthodox	265	85.7
	Muslim	35	11.3
	Catholic	9	3
Marital status	Single	43	13.9
	Married	262	84.8
	Divorced	4	1.2
Educational status of the mother	Primary education	20	6.5
	Secondary education	103	33.3
	Diploma and above	186	60.2
Family size	< 4 individuals	168	54.4
	≥4 individuals	141	45.6
Physical exercise in a day	< 30 min	53	17.2
	≥30 min	256	82.8

### Obstetric and anthropometric characters of the study participants

The prevalence of macrosomia with a birth weight of 4000 g or heavier was 19.1% (95% CI: 14.9, 23). All of the newborns were born at term (37–42 weeks of pregnancy), and 53.7% of them were male. More than two-thirds (68%) of the mothers were primipara, and the majority of the pregnancies (92%) were planned and wanted. More than half (56%) of the mothers were normal weight, and 89.3% of the mothers gained weight ≥ 16 kg. More than three-fifths (65.7%) of the women had a medium dietary diversity score (Table 2).

**Table 2:** Obstetric and anthropometric characteristics of the study participants (*n* = 309)

Variables	Categories	Frequency	Percentage
Parity	Primipara	210	68
	Multipara	99	32
Type of current pregnancy	Unplanned and unwanted	6	1.9
	Wanted but unplanned	19	6.1
	Planned and wanted	284	91.9
Pregnancy interval (years)	< 3 years	81	38.4
	≥3 years	130	61.6
Pre-pregnancy BMI	Underweight	28	9.1
	Normal weight	173	56
	Overweight	76	24.6
	Obese	32	10.4
Total pregnancy weight gain	< 16 kg	276	89.3
	≥16 kg	33	10.7
Birth weight of the newborn	Low birth weight	14	4.5
	Norman birth weight	236	76.4
	Macrosomia	59	19.1
Sex of the newborn	Male	166	53.7
	Female	153	46.3

### Logistic regression analysis of factors associated with childhood stunting

Controlling the potential confounders in the multivariable logistic regression model, giving birth to a macrosomic baby in the last pregnancy, maternal age ≥ 30 years, pre-pregnancy BMI and total pregnancy weight gain ≥16 kg were found to have a positive associations with macrosomia (Table 3).

**Table 3:** Factors associated with macrosomia from logistic regression analysis, Mekelle, Tigray, Ethiopia, 2017

Variables	Categories	Macrosomia		COR (95% CI)	AOR (95% CI)
		Yes (%)	No (%)		
Age	< 30 years	20 (89)	161 (11)	1	1
	≥30 years	39 (69.5)	89 (30.5)	3.5 (1.9, 6.4)	2.6 (1.2, 5.8)*
Family size	< 4 individuals	28 (16.7)	140 (83.3)	1	1
	≥4 individuals	31 (22)	110 (78)	1.4 (0.8, 2.5)	0.4 (0.4, 1.2)
Parity	Primipara	30 (14.3)	180 (85.7)	1	1
	Multipara	29 (29.3)	70 (70.7)	2.5 (1.4, 4.4)	2 (0.7, 5.6)
Weight gain	< 16 kg	44 (15.9)	232 (84.1)	1	1
	≥16 kg	15 (54.5)	18 (45.5)	4.4 (2, 9.4)	11 (3, 37) ***
Pre-pregnancy BMI	Underweight	0	28 (100)	1	1
	Normal weight	22 (12.7)	151 (87.3)	1	1
	Overweight	21 (27.6)	55 (72.4)	2.6 (1.3, 5)	5 (2, 13)**
	Obese	16 (50)	16 (50)	6.9 (3, 15.7)	15 (5, 50)***
Previous macrosomia	No	34 (20)	136 (80)	1	1
	Yes	14 (42.4)	19 (57.6)	2.9 (1.3, 6.5)	2.7 (1.1, 7)*

*p-value < 0.05,**p-value < 0.01, ***p –value < 0.0001

The odds of macrosomia in mothers giving birth to a macrosomic baby in their last pregnancy were almost 3 times greater than in mothers who didn’t give birth to a macrosomic baby in their last pregnancy (AOR = 2.7, 95% CI: 1.1, 7). The odds of being macrosomic in babies born from mothers aged over 30 years during pregnancy were 2.6 times greater than the odds of macrosomia in babies from mothers aged < 30 years (AOR =95% CI: 2.6 (1.2, 5.8)).

The odds of being macrosomic in babies born from mothers who gained weight ≥ 16 kg during pregnancy were 11 times greater than the odds of macrosomia in mothers who gained < 16 kg (AOR = 11, 95% CI: 3, 37). Pre-pregnancy BMI was also associated with macrosomia; the odds of being macrosomic in babies born from overweight mothers were 5 times greater than the odds of macrosomia in normal-weight mothers (AOR = 5, 95% CI: 2, 13), and the odds of being macrosomic in babies born from obese mothers were 15 times greater than the odds of macrosomia in babies from normal-weight mothers (AOR = 15, 95% CI: 5, 50).

## Discussion

In this study, an attempt was made to determine the prevalence and associated factors of macrosomia in the study area. Results of this study revealed that maternal age, giving birth to macrosomic baby in the last pregnancy, pregnancy weight gain, and pre-pregnancy BMI were found to be significant predictors of macrosomia. Though some of the risk factors of macrosomia are preventable, there is a worldwide increase in its prevalence. The prevalence of macrosomia in this study was 19.1% (95% CI = 14.9, 23), which was similar to macrosomia level (15.77%) in Iraq [15]. However, this was greater than the incidence in Iran (2.8%), Nigeria (8%), and Chad (7.6%) [16–18]. The variation in the prevalence of macrosomia with an ethnic difference has been documented [18], and the prevalence in these studies seems to suggest a regional variation. It was also higher than the prevalence in the same region of Ethiopia (6.7%) [19]. This difference might be due to the difference in the socio-economic status of the mothers [1, 13, 20] because the previous studies were carried out in public hospitals; whereas, the current study was conducted in the private clinics where mothers with better socio-economic status get the service.

Regarding the possible predictors of macrosomia in this study, the risk of having macrosomia in obese mothers was fifteen times higher than in normal weight mothers. This finding was supported by recent studies that suggested pre-pregnancy BMI as an important predictor of macrosomia [1, 6, 9, 10, 21, 22]. Despite this, more than half (52%) of the mothers were unable to recall their pre-pregnancy weight in this study. Hence, height and weight recorded in the first trimester of pregnancy were used to calculate pre-pregnancy BMI.

In addition, weight gain during pregnancy was significantly associated with fetal macrosomia, and the odds of giving birth to a macrosomic baby in mothers who gained ≥16 kg during pregnancy were 11 times greater than the odds of macrosomia in their counterparts. This was in line with a study by Nkwabong Elie which revealed that the odds of macrosomia in mothers with a weight gain of ≥16 kg is 10.2 times higher than in mothers who gained < 16 kg [1]. It was also supported by the study by Kathy and colleagues [23].

The association between macrosomia in the last pregnancy and current macrosomia was corroborated by the current study, in which the odds of giving birth to a macrosomic baby in mothers with the previous history of giving birth to the macrosomic baby were 7 times greater than the odds of macrosomia in mothers with no history of giving birth to a macrosomic baby. This was similar to the study by Mardani and colleagues, Said and Manji, and Elie Nkwabong, where a history of giving birth to a macrosomic baby was significantly associated with current macrosomia [1, 6, 8].

The association of macrosomia with maternal age was also evident in this study; the odds of being macrosomic in babies born from mothers aged ≥30 kg during pregnancy were 2.6 times greater than the odds of macrosomia from mothers aged < 30 years. This was supported by results from different studies [10, 16, 24]. A study by Said and Manji stated that maternal age ranging from 30 to 39 years was a significant predictor of macrosomia [8].

As a strength of this study, there were no similar studies conducted in the area; and the data were collected by trained midwives; pregnancy-related data were also collected during pregnancy to reduce recall bias. The main limitations of this study were: being a clinic-based study as the results may not be extrapolated to the general population. The causal relationship among the predictors and outcome variable may not also be established due to the natural drawback of the study design we used. As a result, this study will be a foundation for future researchers to apply and conduct robust study designs like cohort and experimental studies in this area to bring results with better validity.

## Conclusion

The incidence of fetal macrosomia in this study was 19.1%. Pre-pregnancy overweight and obesity, pregnancy weight gain> 16 kg, maternal age ≥ 30 years, and giving birth to a macrocosmic baby in the last pregnancy were found to be significant predictors of macrosomia. The factor with the greatest contribution to macrosomia was pre-pregnancy obesity. These findings mainly underline the importance of achieving and maintenance of normal weight in women of childbearing age, and appropriate weight gain in pregnant women.

## Data Availability

All data supporting the results are available with this manuscript.

## Abbreviations

ANC: Antenatal Care
AOR: Adjusted Odds Ratio
BMI: Body Mass Index
BW: Birth Weight
CI: Confidence Interval
IRB: Institutional Review Board
SPSS: Statistical Package for Social Science
WDDS: Women’s Dietary Diversity Score
