# Correction to: Fetal macrosomia and its associated factors among singleton live-births in private clinics in Mekelle city, Tigray, Ethiopia

**DOI:** 10.1186/s12884-019-2688-6

**Published:** 2019-12-24

**Authors:** Freweini Gebrearegay Tela, Afework Mulugeta Bezabih, Amaha Kahsay Adhanu, Kidanemaryam Berhe Tekola

**Affiliations:** Department of Nutrition and Dietetics, School of Public Health, College of Health Sciences, Mekelle University, Tigray, Ethiopia

**Correction to: BMC Pregnancy Childbirth (2019) 19:219**

**https://doi.org/10.1186/s12884-019-2379-3**

Following publication of the original article [1], we have been notified that the name of one author was spelled incorrectly as **Kidanemariam Berhe Tekola**, when the correct spelling is **Kidanemaryam Berhe Tekola.**
